# Optimizing Sensor Deployment for Multi-Sensor-Based HAR System with Improved Glowworm Swarm Optimization Algorithm

**DOI:** 10.3390/s20247161

**Published:** 2020-12-14

**Authors:** Yiming Tian, Jie Zhang

**Affiliations:** 1 College of Information Engineering, Tianjin University of Commerce, Tianjin 300134, China; 2 School of Engineering, Merz Court, Newcastle University, Newcastle upon Tyne NE1 7RU, UK; jie.zhang@ncl.ac.uk

**Keywords:** human activity recognition, multi-sensor data fusion, selective ensemble, glowworm swarm optimization, sensor layout

## Abstract

Human activity recognition (HAR) technology that analyzes and fuses the data acquired from various homogeneous or heterogeneous sensor sources has motivated the development of enormous human-centered applications such as healthcare, fitness, ambient assisted living and rehabilitation. The concurrent use of multiple sensor sources for HAR is a good choice because the plethora of user information provided by the various sensor sources may be useful. However, a multi-sensor system with too many sensors will bring large power consumption and some sensor sources may bring little improvements to the performance. Therefore, the multi-sensor deployment research that can gain a tradeoff among computational complexity and performance is imperative. In this paper, we propose a multi-sensor-based HAR system whose sensor deployment can be optimized by selective ensemble approaches. With respect to optimization of the sensor deployment, an improved binary glowworm swarm optimization (IBGSO) algorithm is proposed and the sensor sources that have a significant effect on the performance of HAR are selected. Furthermore, the ensemble learning system based on optimized sensor deployment is constructed for HAR. Experimental results on two datasets show that the proposed IBGSO-based multi-sensor deployment approach can select a smaller number of sensor sources while achieving better performance than the ensemble of all sensors and other optimization-based selective ensemble approaches.

## 1. Introduction

Wearable sensor-based human activity recognition (HAR) systems have gained incredible popularity in many human-centered applications such as assisted living [1], intelligent interactive applications [2], athletic activities training [3,4] and factory workers monitoring [5]. Through sensors-based HAR system, accurate and reliable information of people’s activity can be provided for ensuring a safe and sound living environment [6]. Compared with video-based HAR systems, sensor-based HAR systems, which are mainly based on sensing technologies, microelectronics and wireless communication technologies, have more advantages. Video-based HAR systems are not practical in many indoor environments especially when illumination and privacy are considered. In addition, video-based approaches only monitor users in the camera’s specific area. Sensor-based HAR is a challenging but promising research area which has been drawing the attention of researchers from the community of ubiquitous computing, machine learning, medical and healthcare.

Recently, considerable research of HAR has demonstrated the potential of multi-sensor fusion for wearable activity recognition [7,8]. In general, placing various sensors on multiple body parts can allow to obtain richer activity information, which could help improve the performance and robustness of HAR. However, in many real-world applications, it is not practical to deploy numerous sensors on multiple positions of the body. This will not only increase the equipment costs but also bring obtrusions for elderly users, especially those who can live independently. Moreover, as the sensor-based HAR applications typically require a large amount of data to be processed, arranging more sensors will increase the communication burden and consequently the power consumption. Last but not the least, the contribution of each sensor depends on the application and the type of activities to be recognized. Some sensors may bring limited improvement on the recognition performance while increasing the amount of data processing [9]. Therefore, a research-worthy problem for the multi-sensor fusion-based HAR is how to evaluate the performance of sensor nodes and minimize the number of sensors required to HAR to realize the overall configuration optimization of the multi-sensor system.

Generally speaking, in terms of the data processing level of abstraction, multi-sensor fusion strategies can be mainly categorized into three types: data-level fusion [10], feature-level fusion [11] and decision-level fusion [12]. As the lowest level of abstraction, data-level fusion combines the raw data from the multiple wearable sensors directly. A large number of parameters, such as the number of sources, sampling rate and sensing synchronization, are affected by the design choices of sensor fusion method. Considering the system energy consumption, most techniques transform data after extracting features from the sensor node as there is limited number of activities to recognize. Feature-level fusion creates a new high-dimension feature vector based on features from different sensor nodes. Feature selection algorithms are typically useful in the pattern recognition and machine learning community. It is difficult to optimize the layout of sensors in the feature-level fusion because this will change the dimension of the newly created feature vector. Among these three fusion levels, decision-level fusion outputs a unique decision according to the local decision of multiple (homogeneous or heterogeneous) sensors. The main advantages of decision-level fusion include communication bandwidth saving and improved decision accuracy. Therefore, there are many multi-sensor-based HAR studies that have focused on optimizing the decision-level fusion process of multi-sensors.

Over the years, ensemble learning has demonstrated great potential for the improvement of many real-world applications [13,14]. The main idea of ensemble learning is to combine multiple base learners to enhance performance. Ensemble learning builds a classification model in two steps. The first step is to establish a set of basic classifiers. In the second step, the decision information of each basic classifier is merged to give the final decision of the ensemble using a combiner function. However, the improvement of ensemble learning system is not proportional to the number of base classifiers. There would be 2*^M^* − 1 nonempty base classifier subsets if a classifier pool contains *M* base classifiers. This makes selecting a subset of the classifier with the optimal performance an NP-complete problem.

Selective ensemble, which is also known as ensemble pruning, is an approach for extracting a subset of classifiers that optimizes the performance of ensemble learning system. Ensemble pruning approaches can be categorized into three main groups: ordering-based [15,16], optimization-based [17], and clustering-based [18] pruning approaches. Ordering-based selection is first based on an evaluation measure (or criterion) that ranks every classifier and then aggregates the ensemble members whose ranks are above a predefined threshold. Clustering-based pruning approaches consists of a clustering technique, which allows identifying a set of representative classifiers that compose the pruned ensemble. Although these methods have less computational cost, the ensemble performance may not be optimal. In addition, for the above two approaches, there is no ideal way to choose the scale of the ensemble learning system. Comparatively, optimization-based approach treats ensemble pruning as an optimization problem, which makes it easier to obtain the optimal ensemble. In addition, there are many heuristic algorithms that can be used as a searching strategy to find the optimal sub-ensemble, such as the genetic algorithm (GA) [19], particle swarm optimization (PSO) [20], ant colony optimization (ACO) [21] and glowworm swarm optimization (GSO) [22]. However, there are few studies [23] that applied the effective optimization-based pruning approach to design the layout of the multi-sensor for HAR system.

In this study, we design a multi-sensor-based HAR framework which combines the advantages of decision-level fusion and selective ensemble to address the aforementioned challenges. In this proposed HAR framework, the selective ensemble approaches can be applied to reduce the scale of ensemble sensors and improve the effectiveness of the decision-level fusion. Furthermore, to improve the search ability and global convergence of the optimization-based approach, we proposed a novel optimization-based selective ensemble approach, which is named the improved binary glowworm swarm optimization (IBGSO) approach, for selecting the most optimal set of base classifiers automatically. In this way, the sensor combination for the most important parts of the body can be optimized, which will boost the performance of the multi-sensor-based HAR system. The proposed system is demonstrated to be able to achieve the balance of HAR performance and the number of sensors. The main contributions of this paper are as follows:

(1) Activity recognition framework: we design a multi-sensor-based HAR framework in which the sensor deployment can be optimized to find a tradeoff between the number of sensors and system performance.
(2) A novel optimization-based selective approach IBGSO: in order to improve the search ability and global convergence, we propose a novel optimization-based selective approach IBGSO for the multi-sensor-based HAR framework. Compared with the other three state-of-the-art optimization-based selective approaches, the proposed IBGSO approach can help us to comprehensively understand the crucial positions and sensors for the performance of HAR.
(3) Experimental evaluation: we conduct extensive experiments and obtain several valuable results that can help researchers make better decisions in utilizing sensors and positions for multi-sensor-based HAR.

## 2. Related Works

Considerable research has demonstrated the potential of body sensor networks (BSNs) in many physical activity monitoring applications. However, since many activity monitoring applications require sophisticated signal processing, feature extraction and recognition algorithms, the design and optimization of BSN still remain a challenging task. For example, the complex sensory data, especially when these data are uncertain or even incomplete, make the majority voting and naive Bayes fusion methods in decision-level unsuitable for HAR. Recently, Chen et al. [24] proposed a new method based on the Dempster–Shafer theory to improve human action recognition by using the fusion of depth camera and inertial sensors. However, this work ignored that there exists an assumption that the hypotheses considered should be exclusive, which is not applicable to HAR. Dong et al. [25] designed a robust and intelligent sensor fusion strategy based on the Dezert-Smarandache theory for HAR. In this framework, the missing data of the involved sensors are treated as ignorant without manual interpolation or intervention. Boutellaa et al. [26] introduced a multi-wearable sensor-based fall detection system which applied the covariance matrix and neighbor classification techniques to process the signals. The covariance matrix-based processing is beneficial for improving the recognition performance and has improved the mean accuracy of fall detection. Guo et al. [27] introduced a hierarchical data fusion model for HAR by using multiple wearable inertial sensors. In this model, two levels, namely basic classification and a fusion layer, were utilized to analyze the information from the multiple sensors. It is shown that using the entropy-based weight and feature selection can reduce the errors in the decision phase.

In addition to the conventional machine learning algorithms, the recently developed ensemble learning has also been proved to be effective for the task of HAR. Chen et al. [13] proposed a novel ensemble extreme learning machine (ELM) algorithm for HAR based on smartphone sensors. To enhance the diversity among each base ELM, the Gaussian random projection is applied in the novel ensemble algorithm to initialize the input weights of base ELMs. Experiments have demonstrated that the proposed algorithm boosts the performance of ensemble learning. Gibson et al. [28] proposed an accelerometer-based fall detection framework that utilizes multiple classifiers to improve the fall detection and diagnostic performance. The multi-classifier system demonstrated significant performance advantages compared with other classification methods. The performance of five types of ensemble classifiers which employed support vector machine (SVM) and random forest (RF) as the base learners were discussed on HAR [29]. The experiments showed that SVM achieved the highest accuracy rate, 99.22%, based on a random subspace ensemble classifier. Chowdhury et al. [30] compared the performance of the custom ensemble model and conventional ensemble machine learning methods on HAR. The results showed that a custom ensemble model using weighted majority voting achieves the best performance.

## 3. Related and Proposed Techniques

### 3.1. Extreme Learning Machine

In this study, ELMs are utilized as the base classifiers to recognize human activity patterns. Owing to the extremely fast learning speed and the good generalization performance, as a feed-forward neural network, the ELM algorithm proposed by Huang et al. [31] in 2006 has been successfully applied for the task of HAR [13]. Moreover, as the parameters of the algorithm are set randomly, the unstable and diverse results of ELM help to improve the recognition performance of the ensemble learning system.

**Figure 1:**
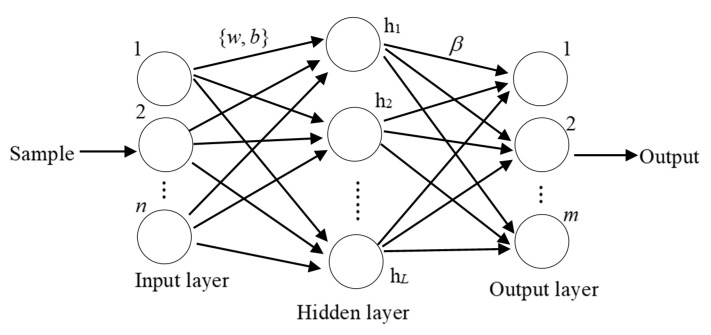
The structure of ELM.

The structure of ELM includes an input layer, a hidden layer and an output layer, which are shown in Figure 1, where *β* is a matrix weights between the hidden nodes and the output nodes and *w* and *b* are the weights and bias from input nodes to hidden node, respectively. ELM is different from the traditional BP learning method, which iteratively updates the parameters of {*w b*} according to the gradient of the modeling error. Traditional BP learning methods are not only time-consuming but also have low generalization performance. The ELM algorithm generates the input weights *w* and bias *b* randomly and provides a closed-form solution of the output layer weights using the least-squares method.

### 3.2. Multi-Sensor Fusion with an Ensemble Learning System

**Figure 2:**
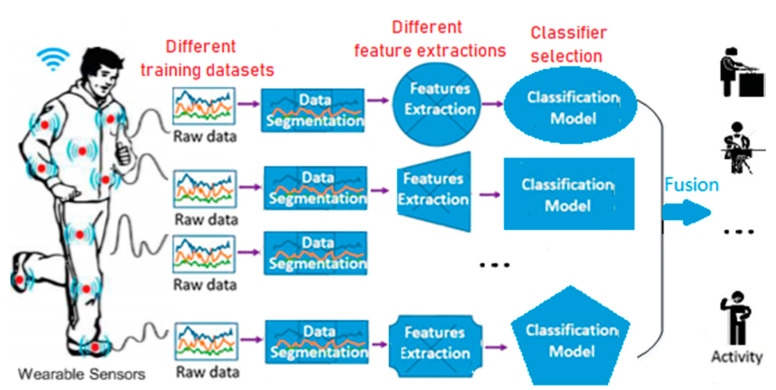
The structure of multi-sensor fusion with an ensemble learning system.

Most works on multi-sensor fusion are based on decision-level fusion. Compared with the other two fusion methods, decision-level fusion can process heterogeneous sensor information with less communication bandwidth consumption. Correspondingly, this paper is based on the decision-level fusion method, which combines the information of several simple classifiers and establishes an ensemble system that includes a one-to-one relationship between classifiers and sensors. The designed structure of multi-sensor fusion with ensemble learning system is shown in Figure 2.

The core idea of ensemble learning is to combine multiple base learners to improve the performance. For base classifier generation, an important principle is the diversity among base classifiers, which can improve the performance of an ensemble system by mining salient information with respect to different perspectives. Generally, there are three approaches to generate the basic classifiers with diversity in the ensemble learning system, as shown in Figure 2. The first is to train the base classifiers with different training datasets such as Bagging, Boosting and random subspace. The second is to construct base classifiers with different feature sets. The last one is to enhance the diversity among base classifiers and performance of the ensemble system by an objective function or evaluative criteria, such as some effective pruning approaches [15,16,17,18].

Base on the approaches mentioned above, this work utilizes two of the above approaches. In the base classifier generation phase, the training of base ELM classifier is realized by data from different body positions, which makes each position on the body correspond to a base classifier in the ensemble learning system. After that, we applied the proposed selective ensemble approach IBGSO to find the optimal set of classifiers to optimize the dense multi-sensor deployment. In this phase, the sensor layout optimization problem is transformed into an ensemble pruning problem. With the optimization of the ensemble learning system, we can utilize the proposed approach to find the optimal sensor layout.

### 3.3. The Proposed Optimization-Based Selective Approach IBGSO

#### 3.3.1. Glowworm Swarm Optimization

GSO [32] is an intelligent optimization algorithm, which is based on the phenomenon that the light emitted by glowworms can be used as a signal to attract other glowworms. The algorithm contains a set of glowworms that are randomly distributed in the solution space. Each glowworm is a possible solution represented by its position. The glowworms with high luminosity have higher brightness, which can attract low-brightness glowworms. In this way, the global optimization of the algorithm can be achieved. The basic steps are as follows.

Step 1. Initialize the basic parameters of the GSO. These parameters include population size *g*, fluorescein volatilization factor *ρ*, fluorescein update rate *γ*, update rate *β* of the dynamic decision domain, the set of glowworms *N_i_*(*t*) in the decision domain, threshold *n_t_* for the number of glowworms in the neighborhood, perception radius *r_s_* and move step *s*.

Step 2. The fitness value of the glowworm *i* at the *t*th iteration is converted into the fluorescein value with the following formula:

(1) li(t)=(1−ρ)li(t−1)+γJ(Xi(t))

where *ρ* is the fluorescein decay constant belonging to (0, 1) and *γ* is the fluorescein enhancement constant.

Step 3. Each glowworm selects individuals with higher brightness than itself within its dynamic decision radius $r_{d}^{i}\left(t\right)$ to form its neighbor set *N_i_*(*t*).

Step 4. Calculate the probability *p_ij_*(*t*) of the glowworm *X_i_*(*t*) moving to the glowworm *X_j_*(*t*) in its dynamic decision radius by Equation (2):

(2) $$p_{ij}(t)=\frac{l_{j}(t)-l_{i}(t)}{\sum_{k\in N_{i}(t)}l_{k}(t)-l_{i}(t)}$$

Step 5. Update the position of glowworm *X_i_*(*t*) by Equation (3):

(3) $$X_{i}(t+1)=X_{i}(t)+s\times \left[\frac{X_{j}(t)-X_{i}(t)}{\left\|X_{j}(t)-X_{i}(t)\right\|}\right]$$

Step 6. Update the dynamic decision radius of the glowworm *X_i_*(*t*) by Equation (4):

(4) $$r_{d}^{i}(t+1)=\min\left\{r_{s},\max\left\{0,\beta \times (n_{t}-\left|N_{i}(t)\right|)\right\}\right\}$$

#### 3.3.2. IBGSO

In order to make the GSO applicable for solving the selective ensemble problem and improve the search ability of the algorithm, this paper proposes IBGSO. Firstly, the movement of glowworms is improved so that GSO can search in a binary discrete space. Secondly, the search behavior of GSO is modified, which can increase the randomness of the algorithm and ensure that the algorithm avoids falling into the local optimum. Finally, mutation behavior is introduced to increase the diversity of the population and improve the search efficiency of the algorithm. These improvements are detailed as follows:

(a) Bulletin board

A bulletin board is added to the algorithm to record the best position and corresponding fitness value in the iterative process of the algorithm. After each iteration of the algorithm, the bulletin board will be updated if the best fitness value in the population is better than the best value in the bulletin board.

(b) Improvement of steps

The GSO does not have the ability to search for the optimal solution in a binary space. Thus, to deal with a discrete combinatorial optimization problem, the move method of the glowworm should be changed. In this paper, we attempt to change the position of glowworms using probability. In the *t*th iteration of the proposed IBGSO algorithm, let $x_{i}(t)=[x_{i1}(t),x_{i2}(t),\cdots ,x_{in}(t)]$ be the position of the current glowworm and $x_{j}(t)=[x_{j1}(t),x_{j2}(t),\cdots ,x_{jn}(t)]$ be the position of the target glowworm that *x_i_*(*t*) will move to. When the position update is performed, the status of the position is changed according to a certain probability. The position update can be expressed mathematically as:

(5) $$x_{ik}(t+1)=\left\{ \begin{array}{l} x_{ik}(t),r\leq p_{1}\\ x_{jk}(t),p_{1}<r<p_{2}\\ r_{0},r\geq p_{2} \end{array}\right.$$

where *p*_1_ and *p*_2_ ∈ [0, 1] are both selected parameters for the update formula, *r* is a random number between (0, 1) and *r*_0_ is the a random number of 0 or 1, *k* = 1, 2, …, *n*.

(c) Improvement of search behavior

In order to improve the convergence speed and the performance of the algorithm, this paper improve the search behavior as follows. In the *t*th iteration, the glowworm *x_i_*(*t*) respectively moves to the best position in the bulletin board, the optimal position of glowworm in the decision domain and a random position in the decision domain. These positions are marked as *x^’^_i_*(*t +* 1), *x^’’^_i_*(*t +* 1) and *x^’’’^_i_*(*t +* 1). Then, the best one of the *x^’^_i_*(*t +* 1), *x^’’^_i_*(*t +* 1) and *x^’’’^_i_*(*t +* 1) will be the position of *x_i_*(*t +* 1).

(d) Mutation behavior

In the iterative process, if the glowworms population gathers seriously at the local optimal value point, the algorithm will easily be trapped in a local optimum, which will affect its convergence. In order to improve the diversity of the GSO algorithm and overcome the problem of premature convergence, this paper introduces the mutation operation, which is described as in Formula (6):

(6) $$x_{id}=\left\{ \begin{array}{l} \tilde{x_{id}}\;\mathrm{if}\;\mathrm{rand}()\leq R\\ x_{id}\;\mathrm{otherwise} \end{array}\right.$$

In the formula, *R* represents the probability of mutation and rand() is a random number uniform distributed between (0, 1). The heuristic algorithm is required to have a strong global search ability in the early stage and a strong local search ability in the later stage. Based on the above consideration, the early mutation rate of the proposed algorithm should be larger, and the late mutation rate should be smaller. Therefore, a decreasing strategy is adopted in this paper, as described in Formula (7):

(7) $$R={\left(1-\frac{t}{t_{\max}}\right)}^{2}$$

In the formula, *t* is the index of the current iteration and *t*_max_ is the maximum number of iterations.

**Figure 3:**
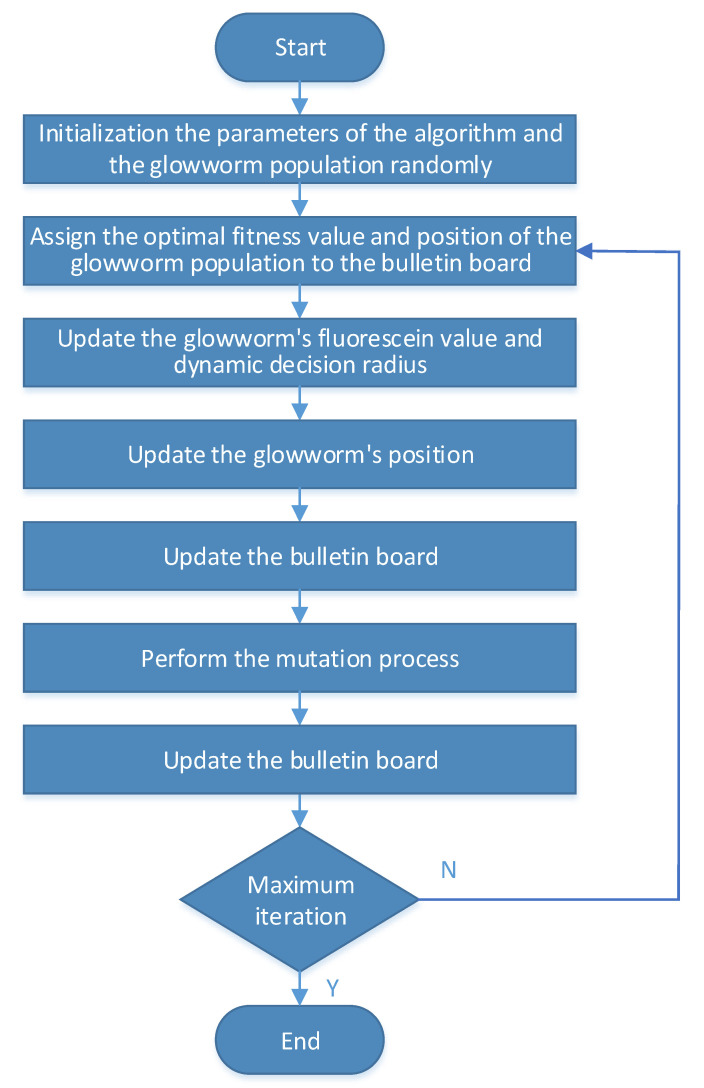
Flowchart of the proposed improved binary glowworm swarm algorithm.

The flowchart of the proposed improved binary glowworm swarm algorithm is shown in Figure 3.

## 4. Optimizing the Sensor Deployment Based on the Proposed IBGSO Selective Ensemble Approach

**Figure 4:**
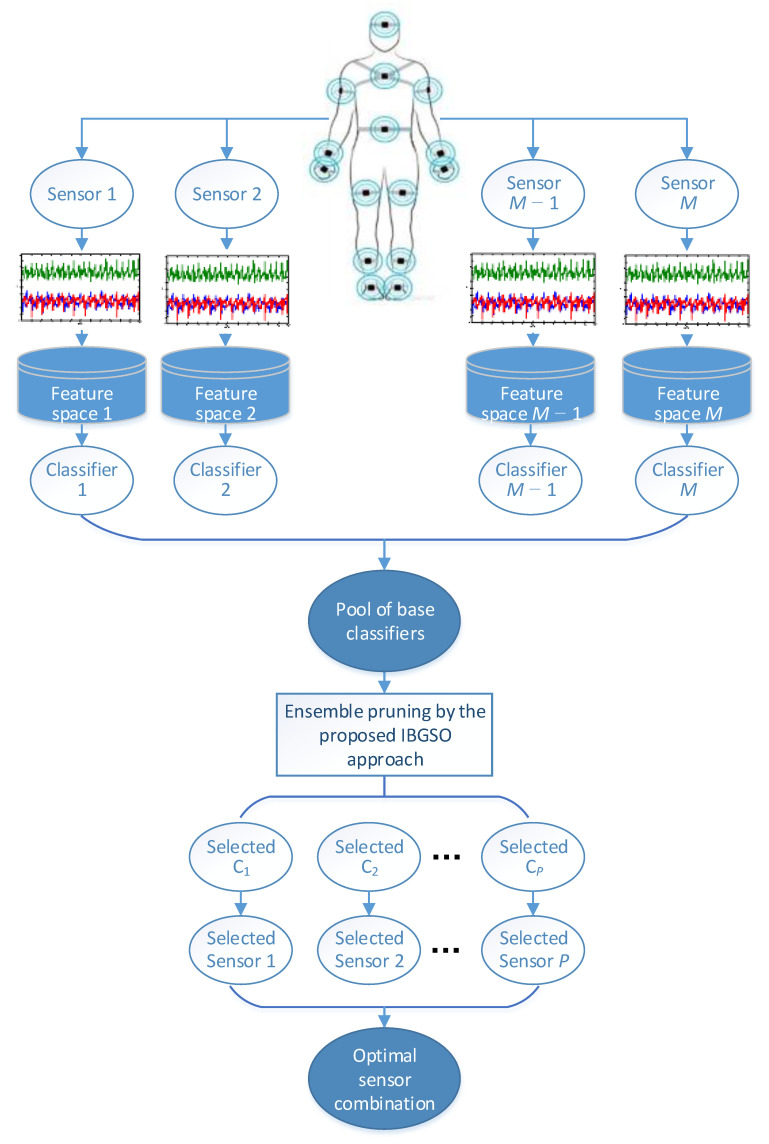
The framework of multi-sensor-based human activity recognition (HAR) with an improved binary glowworm swarm optimization (IBGSO) selective ensemble.

Based on selective ensemble learning and multi-sensor fusion on decision-level, this study proposes a sensor layout optimization scheme for HAR and a corresponding ensemble pruning approach. The proposed HAR structure can make use of the information from multiple body positions and find the optimal sensor subset according to the required performance of the system adaptively. The designed framework of multi-sensor-based HAR with a selective ensemble is specifically illustrated in Figure 4.

As shown in Figure 4, we have established a one-to-one mapping relationship between sensors and classifiers, while the sensor layout optimization problem can be regarded as a selective ensemble problem for the ensemble learning system. The optimal classifier set is selected by the proposed IBGSO approach and the corresponding optimal sensor position can be determined. The proposed method mainly includes the following steps:

(1) Obtain the feature set of each activity from different positions. In consideration of the requirements of the performance and efficiency of the HAR system, in this work, the maximum, minimum, mean value, root mean square, standard deviation *σ*, skewness *S*, kurtosis *K* and the signal energy *E* are utilized as feature construction. Some of these features can be expressed as follows:
  (8) $$mean=\frac{1}{N}\sum_{i=1}^{N}a_{i}$$
  (9) $$\sigma =\sqrt{\frac{1}{N}\sum_{i=1}^{N}{\left(a_{i}-mean\right)}^{2}}$$
  (10) $$K=\frac{1}{N}\sum_{i=1}^{N}{\left(a_{i}-mean\right)}^{4}/\sigma^{4}$$
  (11) $$S=\frac{1}{N}\sum_{i=1}^{N}{\left(a_{i}-mean\right)}^{3}/\sigma^{3}$$
  (12) $$E=\sum_{i=1}^{N}{\left|a_{i}\right|}^{2}$$
  (13) $$RMS=\sqrt{\frac{1}{N}(a_{1}^{2}+a_{2}^{2}\cdots +a_{N}^{2})}$$
  where *a_i_* is the acceleration data, *i* = 1, 2, …, *N*. *N* is the number of data points. After feature extraction, all features were normalized to the interval [0, 1] to eliminate the impact of the range difference.
(2) Generate various individual classifiers. The activity data corresponding to the different positions of the body is employed to initially establish the ELMs. Moreover, the aggregating concept is utilized to combine the trained base ELMs. In this work, the ensemble learning model for HAR is, thus, built with multiple basic classifiers corresponding to positions and we utilize the majority voting method to fuse the decision information of different positions of the body.
(3) Select the optimal subset of ELMs by the proposed IBGSO method.
  After the IBGSO parameter initialization, the optimization process for the optimal ensemble subset begins. This work utilizes a binary encoding method (a combination of 0 and 1), which can represent the state of the base ELMs selection. Let binary strings $C=\{c_{1},\;c_{2},\;\cdots ,\;c_{M}\}$ express the original base ELMs ensemble and *M* be the number of ELMs. If *c_i_* = 1, then it represents that the *i*th base ELM is selected; if *c_i_* = 0, it indicates that the *i*th base ELM is not selected. Therefore, the modified IBGSO algorithm can deal with the selective ensemble. For each glowworm, the bits in the binary strings can represent whether the base ELMs corresponding to the poisons will be selected.
  The sensor layout is optimized to reduce the placement of sensors and improve the performance of the multi-sensor motion recognition system. Therefore, when evaluating the sensor layout, their recognition accuracy is taken as an important reference factor in this work. In addition, we take the scale of the ensemble system (that is, the number of sensors) as another secondary optimization goal, so we introduce a new fitness function as follows:
  (14) $$fitness=\omega \times A_{tr}-(1-\omega )\frac{m}{M}$$
  where *A_tr_* is the training accuracy, *M* is the total number of ELMs, *m* is an integer that satisfies 0<m≤M and represents the number of selected ELMs and *ω* is a weighting factor which is slightly less than 1. If the two ensemble subsets have the same accuracy, the ensemble subset with fewer base ELMs will have a lager fitness value. For each glowworm, the fitness value is calculated as the function (14) and the base classifier combination corresponding to the maximum fitness function will be obtained.
(4) Employ the selective ensemble system with optimized sensor layout to HAR
  The proposed HAR method combines multiple classifiers, which are constructed by activity data from different body positions. Moreover, through the proposed optimization-based classifier selection approach IBGSO, we can reduce the number of sensors and ensure that the system has better recognition performance. Therefore, the proposed HAR method has high practicability, which can realize the optimal performance of multi-sensor system with a minimum number of sensors.

## 5. Datasets and Experimental Setup

### 5.1. Datasets

We utilized two real activity recognition datasets, which were collected from on-body sensors. The first dataset contains various sensor nodes on the body positions, which is beneficial for our work for the variety of the optimization performance of the sensor layout. The second dataset has many kinds of activity; we can utilize this dataset to check the HAR performance of our work. The details of the datasets are described as described below.

The OPPORTUNITY dataset contains the data of human daily activities recorded by 72 sensors of 10 modalities, integrated with the environment, in objects and on the body. The details of the sensor dataset can be found in the OPPORTUNITY UCI dataset. In this work, we only utilized the data acquired from wearable sensors of body sensor networks, which consisted of 7 inertial measurement units (IMU) sensors and 12 3-axis accelerometers. The sensor types and their body positions utilized in this work are shown in Table 1. There are various sensor types in the table, which include an accelerometer (Acc), gyro (Gyro), magnetic (Magn), quaternion (Quat), Eu, Nav, Body, AngVelBodyFrame and AngVelNavFrame. For the processing of missing data in this dataset, we set the data recordings to zero if more than half the data were missing and copied the previous data if a small piece of the data was missing. We utilized this dataset to recognize four kinds of daily activities including standing (A1), walking (A2), sitting (A3) and lying down (A4), which were performed by the four subjects five times. Then, the sliding window of 0.5 s was utilized to divide the signal and a 50% overlap between adjacent windows was adopted.

Daily and sports activities dataset (DSA). This dataset consists of multi-sensor activity data from the torso (T), right arm (RA), left arm (LA), right leg (RL) and left leg (LL) collected by the MTx unit. Each MTx unit has a tri-axial accelerometer, a tri-axial gyroscope and a tri-axial magnetometer. The sensor types and their body positions are shown in Table 2. The dataset has 19 kinds of activities, which are shown in Table 3. Each activity listed in Table 3 is performed by eight volunteer subjects (four female, four male; ages 20–30) for 5 min. The MTx sensor units are calibrated to acquire data at 25 Hz sampling frequency. The 5 min signals are divided into 5 s segments so that 480 (60 × 8) signal segments are obtained for feature extraction.

### 5.2. Performance Evaluation

The performance evaluations implemented in this study are briefly introduced as follows:

Accuracy is calculated as the ratio of the number of samples correctly classified by the classifier and the total number of all the samples and can measure the overall performance of a classifier as shown in Equation (15):

(15) $$\mathrm{Accuracy}\;=\frac{TP+TN}{TP+TN+FP+FN}$$

where the variables *TP*, *TN*, *FP* and *FN*, respectively, represent the number of true positive, true negative, false positive and false negative outcomes in a given experiment. In addition, F1 evaluation criteria are also considered and are defined as the combination of the precision and the recall. Precision represents the proportion of true positive samples among all samples classified as positive. Recall represents the proportion of all the positive samples that are classified as positive samples. The precision, recall and F1 are calculated as follows:

(16) $$\mathrm{precision}\;=\frac{TP}{TP+FP}$$

(17) $$\mathrm{recall}=\frac{TP}{TP+FN}$$

(18) $$F1=\frac{2\;\times \;\mathrm{recall}\;\times \;\mathrm{precision}}{\mathrm{recall}\;+\;\mathrm{precision}}$$

### 5.3. Experiment Setup

In this work, we utilized the subject-based leave-one-out cross validation method to verify our proposed HAR approach. This approach takes a different subject out for testing in each repetition and the data from the rest subjects are utilized for training until all the data from subjects have been utilized for testing. The parameters of IBGSO are set as follows: fluorescein volatilization factor *ρ* = 0.4, fluorescein update rate *γ* = 0.6, dynamic decision domain update rate *β* = 0.08, threshold *n_t_* = 5 and the maximum number of iterations *t*_max_ = 300, *p*_1_ = 0.15 *p*_2_ = 0.85.

## 6. Experimental Results

In order to verify the effeteness of the proposed approach for optimizing the sensor layout of the multi-sensor HAR system, we compared it with the following algorithms: GA (Genetic algorithm) [33], BAFSA (Binary artificial fish swarm algorithm) [34] and BGSO (Binary glowworm swarm optimization) [35]. All of the aforementioned heuristic algorithms are binary searching algorithms. The maximum number of iterations and the population size for these four algorithms are all set to the same.

### 6.1. Experiment 1: OPPORTUNITY Dataset

Table 4 and Table 5 show the sensor selection results of the randomly selected two subjects, respectively, by utilizing the proposed and the comparative approaches. For each subject, we perform the experiment five times to scientifically verify the effectiveness of the proposed approach. It is apparent that the number and the set of classifiers (sensors) selected by the four algorithms differ with subjects. Among the results of the four algorithms, the number of classifiers (sensors) selected by IBGSO approach is between 9 and 13. GA selects 13 to 16 classifiers (sensors). Compared with the GA, the BAFSA and BGSO approaches normally decreases on average 2 to 4 sensors and 3 to 5 classifiers (sensors), respectively. For a specific subject, the number of the classifiers (sensors) obtained by the proposed IBGSO approach is the smallest, which demonstrates that the proposed approach has a strong search ability and can reduce the ensemble scale of base classifiers (sensors) effectively.

**Figure 5:**
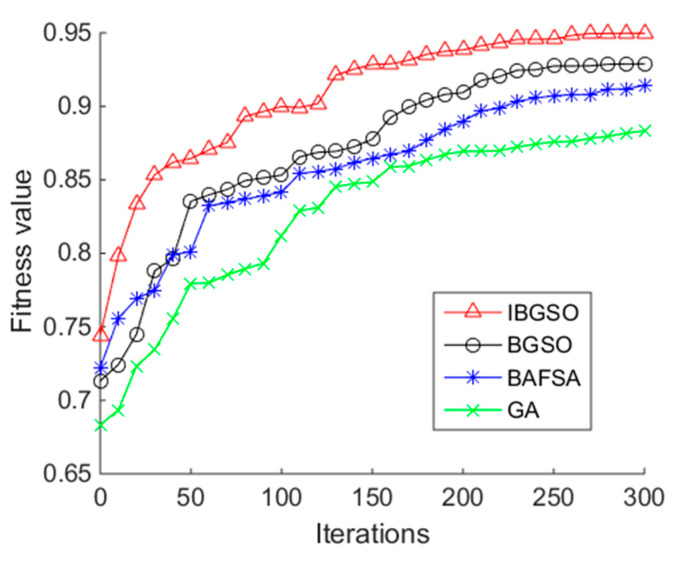
Relationship between the fitness value of the heuristic algorithms and the iterations using the OPPORTUNITY dataset.

Figure 5 shows the fitness evolutionary curves of the four heuristic algorithms. From Figure 5, it can be seen that the proposed IBGSO has better convergence and search performance than the other three binary heuristic algorithms overall. In the early phase of the algorithm iteration, the proposed algorithm has a rapid improvement of fitness value compared with the other three algorithms. In the middle phase of the algorithm iteration, the fitness value slowly increases and until the latter phase of the iteration, the fitness curve, levels off. After the 250th iteration, the fitness value of IBGSO cannot be significantly improved.

According to the sensor selection results of the four optimization approaches, Table 6 and Table 7 respectively show their average testing performance, including the accuracy and F1 of the four subjects. It can be seen from Table 6 and Table 7 that the set of classifiers (sensors) obtained by the proposed IBGSO approach can attain the best performance among all the approaches on most subjects, except subject 2. The performance achieved by IBGSO for subject 2 is slightly less than the ensemble of all approaches, but the number of classifiers (sensors) utilized by IBGSO is significantly less than the ensemble of all approaches; this will be described in more detail below. For a lightweight and robust multi-sensor system, it is worthwhile to achieve a more ideal recognition performance with fewer sensors. In addition, the results show that the ensemble of all the classifiers (sensors) fail to obtain the best performance on most subjects, which demonstrates that using all classifiers (sensors) may not give the best system performance.

Table 8 shows the average testing performance for all subjects with regard to the accuracy, F1 and the ensemble size; it can be seen from Table 8 that IBGSO has a better performance than the other three approaches using a lower number of classifiers (sensors). For the proposed IBGSO, more than 75% of the classifiers (sensors) are pruned. From the results of the ensemble size and performance, it can be seen that the four optimization approaches differ greatly in the number of selected classifiers (sensors) and performance; the number of sensors is not the only condition for improving recognition performance. The proposed IBGSO-based approach helps us to optimize the sensor layout and achieve the trade-off between the number of sensors and the average performance.

**Figure 6:**
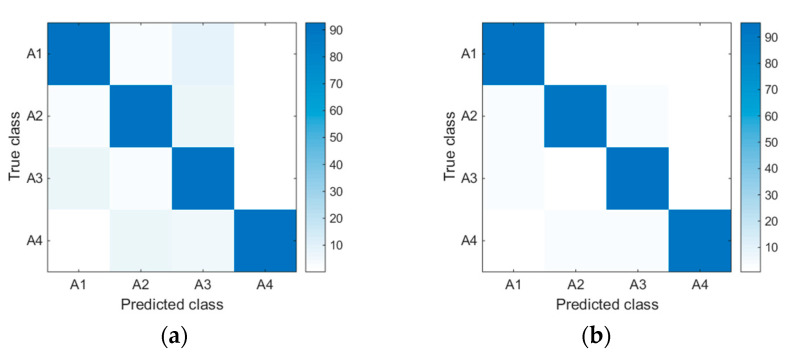
Confusion matrices for the ensemble all approach (**a**) and the proposed IBGSO-based ensemble approach (**b**) using the OPPORTUNITY dataset.

Additionally, the confusion matrixes for the ensemble of all approaches and the proposed IBGSO-based ensemble are constructed in Figure 6 to show a better insight into the effectiveness of the proposed method in optimizing sensor deployment. Figure 6a shows the result of the ensemble of all approaches and Figure 6b shows the results of the proposed approach. It can be seen from the comparison that there are major confusions among activities, such as A1 and A3, A2 and A3 and A2 and A4 in the ensemble of all approaches, while the proposed IBGSO-based approach increases the discrimination of these activities. Therefore, the HAR method proposed in this paper can effectively reduce the number of sensors and improve the recognition performance.

### 6.2. Experiment 2: Daily and Sports Activities Dataset

In this dataset, as each direction component of the three kinds of sensors (accelerometer, gyroscope, magnetometer) is regarded separately as a sensor (Table 2), we can find the optimal set of the sensor orientation components using data from five body parts with the proposed IBGSO approach. Table 9 and Table 10 respectively show the combination of sensor orientation components that have an important impact on the HAR performance, obtained by using the four optimization approaches based on two randomly selected subjects. For each subject, we performed the experiment five times. It can be seen from the Table 9 and Table 10 that the size of the classifiers (sensors) obtained by the proposed IBGSO approach is between 13 and 16, which is the smallest compared with the other three algorithms. This result is similar to the OPPORTUNITY dataset. Moreover, it can be seen that no matter which approach is adopted, the components of the tri-axial accelerometer from the torso (T) are always selected. However, the components of the tri-axial accelerometer from other positions are not. Therefore, the tri-axial accelerometer on the torso is recommended to be deployed in a multi-sensor-based system for HAR.

**Figure 7:**
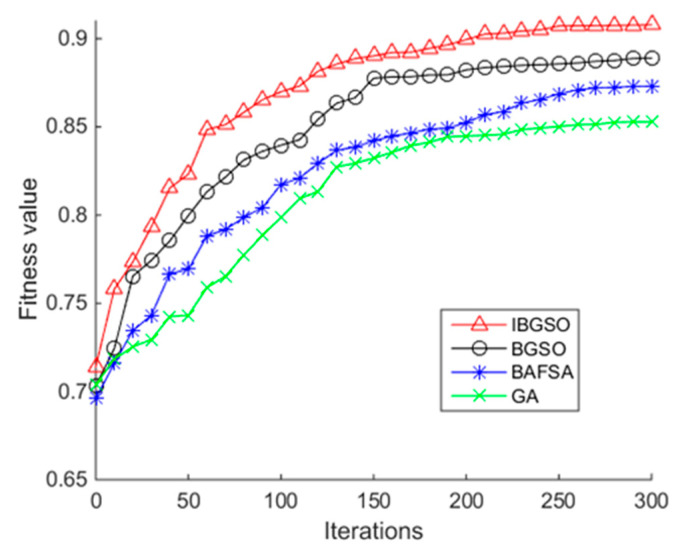
Relationship between the fitness value of the heuristic algorithms and iterations using the DSA.

Figure 7 shows the fitness evolutionary curves of the four heuristic algorithms when the daily and sports activities dataset is utilized. It can be seen from Figure 7 that the fitness values of the four optimization algorithms are improved with the iteration times and the proposed IBGSO produces a similar performance trend to Figure 5 during the iteration process. Overall, the proposed IBGSO is superior to the other three algorithms in terms of convergence and search performance.

Based on the selection results of the sensor properties and the direction components from five body positions, the average testing performances of the four optimization approaches for the four randomly selected subjects are shown in Table 11 and Table 12. It can be seen that the proposed IBGSO can attain the best performance among all the approaches on the randomly selected subjects. Therefore, this demonstrates that the proposed IBGSO approach is also effective when applied to the daily and sports activities dataset.

The ensemble sizes and the testing performances of the four algorithms are shown in Table 13 when the daily and sports activities dataset is utilized. It can be seen that IBGSO selects the lowest number of classifiers (sensors), while GA uses the highest number of classifiers (sensors) among the three optimized approaches. Moreover, IBGSO has better performance than the other three approaches using a smaller number of classifiers (sensors). For the daily and sports activities dataset, more than 70% of the classifiers (sensors) are pruned by using IBGSO. Therefore, it can be concluded that IBGSO is also the optimum approach on this dataset.

**Figure 8:**
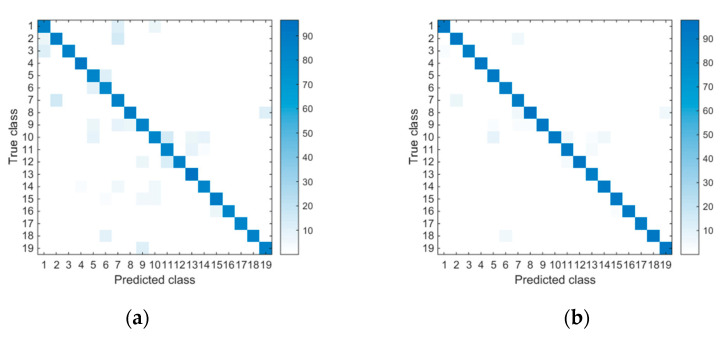
Confusion matrices for the ensemble all approach (**a**) and the proposed IBGSO-based ensemble approach (**b**) using the DSA.

Figure 8 shows the confusion matrixes for ensemble all and proposed IBGSO-based ensemble on DSA. From the left matrix, we can see that there are many activities that have been misrecognized. For example, A1 is easily recognized as A9, A5 is easily recognized as A6 and A10 is easily recognized as A11. From the right one, minor confusions can be seen from these activities. Therefore, this demonstrates that the proposed approach for optimizing sensor deployment is also effective for the DSA dataset, which contains various kinds of activities.

## 7. Conclusions

This paper addresses the challenge of the HAR problem in the multi-sensor network from the perspective of optimization sensor deployment to gain a tradeoff among computational complexity and performance for a multi-sensor-based HAR system. We designed a multi-sensor-based HAR framework and proposed a novel optimization-based selective approach IBGSO to select the most crucial positions and sensors for HAR. Extensive experiments on two wearable sensor-based HAR datasets (OPPORTUNITY dataset and DSA) demonstrated the superiority of the proposed approach. For these two datasets, respectively, 0.926 accuracy and 0.946 F1 with an average of 10.8 sensors and 0.842 accuracy and 0.875 F1 with an average of 13.4 sensors. The representative sensors selected by the proposed optimization-based selective approach IBGSO have the advantages of a smaller number of classifiers and better performance compared with other optimization-based approaches.

In future work, we will explore the performance of the proposed method of the sensor deployment optimization on certain specific activities such as fall or gait, which will have beneficial significance for the clinical diagnosis of some diseases such as stroke and movement disorders. In addition, in the future we will attempt to modify the proposed IBGSO approach and find other heuristic algorithms for optimizing the sensor deployment.

## Figures and Tables

**Table 1:** The sensor types and their body positions utilized in this work.

No.	Position/Type	No.	Position/Type	No.	Position/Type
S1	RKNˆ/Acc	S15	IMU BACK/Magn	S29	IMU LLA/Acc
S2	HIP/Acc	S16	IMU BACK/Quat	S30	IMU LLA/Gyro
S3	LUAˆ/Acc	S17	IMU RUA/Acc	S31	IMU LLA/Magn
S4	RUA/Acc	S18	IMU RUA/Gyro	S32	IMU LLA/Quat
S5	LH/Acc	S19	IMU RUA/Magn	S33	IMU L-SHOE/Eu
S6	BACK/Acc	S20	IMU RUA/Quat	S34	IMU L-SHOE/Nav
S7	RKN_/Acc	S21	IMU RLA/Acc	S35	IMU L-SHOE/Body
S8	RWR/Acc	S22	IMU RLA/Gyro	S36	IMU L-SHOE/AngVelBodyFrame
S9	RUAˆ/Acc	S23	IMU RLA/Magn	S37	IMU L-SHOE/AngVelNavFrame
S10	LUA_/Acc	S24	IMU RLA/Quat	S38	IMU R-SHOE/Eu
S11	LWR/Acc	S25	IMU LUA/Acc	S39	IMU R-SHOE/Nav
S12	RH/Acc	S26	IMU LUA/Gyro	S40	IMU R-SHOE/Body
S13	IMU BACK/Acc	S27	IMU LUA/Magn	S41	IMU R-SHOE/AngVelBodyFrame
S14	IMU BACK/Gyro	S28	IMU LUA/Quat	S42	IMU R-SHOE/AngVelNavFrame

**Table 2:** The sensor types and their body positions in the daily and sports activities dataset (DSA).

No.	Pos/Typ	No.	Pos/Typ	No.	Pos/Typ	No.	Pos/Typ	No.	Pos/Typ
S1	T_*x*acc	S10	RA_*x*acc	S19	LA_*x*acc	S28	RL_*x*acc	S37	LL_*x*acc
S2	T_*y*acc	S11	RA_*y*acc	S20	LA_*y*acc	S29	RL_*y*acc	S38	LL_*y*acc
S3	T_*z*acc	S12	RA_*z*acc	S21	LA_*z*acc	S30	RL_*z*acc	S39	LL_*z*acc
S4	T_*x*gyro	S13	RA_*x*gyro	S22	LA_*x*gyro	S31	RL_*x*gyro	S40	LL_*x*gyro
S5	T_*y*gyro	S14	RA_*y*gyro	S23	LA_*y*gyro	S32	RL_*y*gyro	S41	LL_*y*gyro
S6	T_*z*gyro	S15	RA_*z*gyro	S24	LA_*z*gyro	S33	RL_*z*gyro	S42	LL_*z*gyro
S7	T_*x*mag	S16	RA_*x*mag	S25	LA_*x*mag	S34	RL_*x*mag	S43	LL_*x*mag
S8	T_*y*mag	S17	RA_*y*mag	S26	LA_*y*mag	S35	RL_*y*mag	S44	LL_*y*mag
S9	T_*z*mag	S18	RA_*z*mag	S27	LA_*z*mag	S36	RL_*z*mag	S45	LL_zmag

**Table 3:** The kinds of activities in the DSA.

NO.	Activity	NO.	Activity	NO.	Activity
A1	Sitting	A8	Moving around	A15	Cycling on an exercise bike in a horizontal position
A2	Standing	A9	Walking in a parking	A16	Cycling on an exercise bike in a vertical position
A3	Lying on back	A10	Walking on a treadmill(4 km/h, flat)	A17	Rowing
A4	Lying on right side	A11	Walking on a treadmill(4 km/h, inclined positions)	A18	Jumping
A5	Ascending stairs	A12	Running on a treadmill (8 km/h)	A19	Playing basketball
A6	Descending stairs	A13	Exercising on a stepper		
A7	Standing in an elevator	A14	Exercising on a cross trainer		

**Table 4:** Sensor selection results for subject 1 with five runs using the OPPORTUNITY dataset.

Run	GA	BAFSA	BGSO	IBGSO
1	1,4,7,9,10,13,16,17,19,20,22,25,27,35,39,40	1,4,6,9,10,12,14,17,19,21,25,27,27,35	1,7,9,13,16,17,20,23,31,37,39	1,7,9,13,16,17,23,25,31
2	1,9,11,13,25,16,18,20,22,23,27,35,38,40,	2,5,9,10,13,16,20,22,25,31,40	1,7,9,12,17,20,23,25,27,29,31,37	1,7,9,13,17,23,29,31,35,37.39
3	1,2,4,5,6,9,16,17,18,21,23,27,29,31,36,39,40,	1,3,7,9,16,17,21,25,28,31,35,38,40	1,5,7,9,12,17,20,22,23,25,27,29	1,5,7,9,13,16,17,21,25,27,37,39
4	1,7,8,12,13,16,17,20,23,28,31,35,39	1,4,6,7,9,10,12,15,17,19,22,24,28,31,40	1,4,5,7,9,16,17,20,23,27,35,37	1,3,5,7,8,16,17,20,23,25,27,35,37
5	2,5,9,12,17,19,21,25,27,28,31,33,36	2,6,9,19,12,14,16,17,21,23,25,27,28,35,37	1,7,9,12,16,17,20,22,31,35,37,39	1,5,7,13,16,17,22,23,27,31,35,37

**Table 5:** Sensor selection results for subject 2 with five runs using the OPPORTUNITY dataset.

Run	GA	BAFSA	BGSO	IBGSO
1	1,2,4,7,10,13,15,17,19,20,23,25,28,29,31,34	2,3,6,7,10,12,13,17,18,19,23,25,29,31	1,3,6,7,10,13,21,25,27,31,35	1,2,3,7,10,13,25,28,29,31
2	1,2,3,7,10,13,17,18,21,25,27,28,29,31,34	1,2,3,7,10,13,17,25,27,29,31	1,7,10,13,17,23,25,27,28,29,31	1,5,7,10,13,17,21,25,27,28,31
3	1,3,6,7,9,12,14,16,18,19,25,27,29,31,35	1,2,3,9,10,16,25,27,28,29,35,37,38	1,3,5,10,12,13,17,19,25,28,29	1,2,3,7,13,25,27,29,31
4	1,2,6,7,10,13,14,16,18,25,27,28,29,31,35	1,2,7,9,10,13,17,21,25,27,29,31,35,38	1,4,7,9,16,17,20,23,27,35	2,3,5,7,10,13,21,27,31,35
5	1,2,4,7,9,10,13,16,19,22,25,27,28,29,31,35,	1,2,4,5,7,9,10,13,16,19,25,27,28,31,34,35	1,3,7,10,13,17,21,25,28,29	1,2,3,10,13,17,25,27,28

**Table 6:** Accuracy comparison of the four subjects for the five approaches using the OPPORTUNITY dataset.

Method	Subject 1	Subject 2	Subject 3	Subject 4
Ensemble all	0.932	0.927	0.912	0.877
GA	0.862	0.861	0.865	0.824
BAFSA	0.918	0.910	0.876	0.864
BGSO	0.907	0.896	0.913	0.892
IBGSO	0.939	0.923	0.926	0.916

**Table 7:** F1 comparison of the four subjects for the five approaches using the OPPORTUNITY dataset.

Method	Subject 1	Subject 2	Subject 3	Subject 4
Ensemble all	0.928	0.937	0.927	0.916
GA	0.911	0.873	0.898	0.866
BAFSA	0.927	0.923	0.948	0.918
BGSO	0.938	0.935	0.937	0.934
IBGSO	0.954	0.929	0.952	0.949

**Table 8:** Performance comparison for the five approaches using the OPPORTUNITY dataset.

Method	Accuracy	F1	Ensemble Size
Ensemble all	0.912	0.927	45
GA	0.853	0.887	15.4
BAFSA	0.892	0.929	13.6
BGSO	0.902	0.936	12
IBGSO	0.926	0.946	10.8

**Table 9:** Sensor selection results for subject 1 with five runs using the DSA.

Run	GA	BAFSA	BGSO	IBGSO
1	1,3,5,6,9,10,12,16,18,19,21,22,23,25,28,37,39,43,	1,2,5,7,10,11,16,17,19,22,28,37,38,40,42	1,2,3,5,10,12,15,19,20,28,30,37,38	1,2,3,5,10,11,17,19,24,29,30,37,40
2	1,3,5,6,7,8,12,13,16,19,21,24,27,30,35,36,38,40,43	1,3,5,7,8,10,13,19,20,22,29,30,37,38,39,42,44	1,2,3,6,10,11,14,19,20,28,29,31,37	1,3,6,7,10,11,19,20,21,28,38,39,42
3	1,2,4,5,6,9,16,17,18,21,23,27,29,31,36,39,40,	1,5,7,6,10,12,13,15,19,22,28,29,37	1,3,5,10,12,16,18,19,20,22,29,31,37,38	1,2,3,5,6,10,12,17,18,20,21,28,29,34,39
4	2,3,7,8,9,12,15,17,20,25,27,29,37,38,40,42,43	1,2,4,5,6,10,12,15,16,19,20,28,37,38,42	1,2,4,5,10,19,20,26,28,30,37,39,42,44	1,3,5,7,9,10,13,15,19,24,29,32,37,39,42
5	1,2,5,6,9,13,15,17,19,22,25,27,28,30,37,38,42,44	1,3,5,8,9,10,13,19,20,23,25,29,31,37,39,40,42	1,2,3,4,9,10,12,19,21,28,29,30,37,38,41,44	1,2,4,7,10,13,19,20,21,22,27,29,37,39,42

**Table 10:** Sensor selection results for subject 2 with five runs using the DSA.

Run	GA	BAFSA	BGSO	IBGSO
1	1,2,3,7,6,9,11,12,17,18,20,21,24,26,27,29,32,37,38,42	1,2,4,7,8,9,10,11,15,18,20,23,27,29,33,37,38,40	1,2,4,5,11,12,13,15,18,20,21,27,31,37	1,2,3,6,7,10,11,16,19,22,26,28,30
2	1,2,3,4,5,7,9,10,12,16,17,20,23,25,27,30,33,35,38,42,43	1,3,6,7,8,9,10,11,14,16,19,20,22,28,29,30,33,38,41,44	1,3,5,8,9,11,13,16,17,20,28,30,33,37,38	1,2,4,6,7,8,10,11,16,19,21,26,29,37
3	1,2,3,4,5,7,10,14,18,19,20,23,26,28,30,35,37,39,40	1,5,8,9,11,15,17,19,20,21,28,29,37	2,3,5,6,7,11,13,15,18,28,29,34,37,39	1,2,3,5,7,9,10,11,12,15,19,21,29,37
4	1,2,5,7,8,9,11,14,17,21,25,23,26,28,29,31,33,35,40,42,43	1,2,3,5,8,10,12,19,21,23,26,28,29,31,37,39	1,2,4,5,10,19,20,26,28,30,37,39,42,44	1,2,3,4,6,9,10,19,21,28,35,37,39
5	1,2,4,6,8,12,16,17,18,21,24,27,29,32,34,35,37,38,42,44	1,3,4,6,8,9,11,17,18,19,22,24,26,28,29,36,38,40,42	1,2,3,4,10,12,19,21,22,28,29,30,37,38,41,44	1,2,5,7,9,10,12,15,21,22,26,28,32,35,37,39

**Table 11:** Accuracy comparison of the four randomly selected subjects for the five approaches using the DSA.

Method	Subject 1	Subject 2	Subject 3	Subject 4
Ensemble all	0.856	0.805	0.816	0.831
GA	0.745	0.675	0.707	0.729
BAFSA	0.775	0.736	0.765	0.752
BGSO	0.821	0.784	0.799	0.764
IBGSO	0.865	0.837	0.818	0.848

**Table 12:** F1 comparison of the four randomly selected subjects for the five approaches using the DSA.

Method	Subject 1	Subject 2	Subject 3	Subject 4
Ensemble all	0.874	0.821	0.836	0.865
GA	0.787	0.702	0.748	0.771
BAFSA	0.842	0.729	0.807	0.762
BGSO	0.864	0.827	0.818	0.819
IBGSO	0.912	0.854	0.842	0.892

**Table 13:** Performance comparison for the five approaches using the DSA.

Method	Accuracy	F1	Ensemble Size
Ensemble all	0.827	0.849	45
GA	0.714	0.752	18.6
BAFSA	0.757	0.785	16.2
BGSO	0.792	0.832	15.8
IBGSO	0.842	0.875	13.4
